# Serum Cotinine Levels and Prehypertension in Never Smokers

**DOI:** 10.1155/2013/284524

**Published:** 2013-02-17

**Authors:** Omayma Alshaarawy, Jie Xiao, Michael E. Andrew, Cecil Burchfiel, Anoop Shankar

**Affiliations:** ^1^Department of Epidemiology, West Virginia University School of Public Health, 1 Medical Center Drive, P.O. Box 9190, Morgantown, WV 26506, USA; ^2^Biostatistics and Epidemiology Branch, Health Effects Laboratory Division, National Institute for Occupational Safety and Health, Centers for Disease Control and Prevention, Morgantown, WV 26505, USA

## Abstract

*Background*. Few studies have shown that self-reported secondhand smoke exposure in never smokers is associated with high blood pressure. However, there are no studies investigating the relationship between secondhand smoke exposure, measured objectively by serum cotinine levels, and high blood pressure in never smokers. *Methods*. We examined never smokers (*n* = 2027) from the National Health and Nutrition Examination Survey 2005–2008. Our exposure of interest was the secondhand smoke exposure estimated by serum cotinine level and our outcome was prehypertension (*n* = 734), defined as a systolic blood pressure of 120–139 mmHg or diastolic blood pressure of 80–89 mmHg. *Results*. We found that, in never smokers, serum cotinine levels were positively associated with prehypertension. Compared to those with cotinine levels in the lowest quartile (≤0.024 ng/mL), the multivariable odds ratio (95% confidence interval) of prehypertension among those with cotinine levels in the highest quartile (≥0.224 ng/mL) was 1.45(1.00, 2.11); *P* trend = 0.0451. In subsequent subgroup analyses, the positive association was found to be stronger among men, non-Whites, and non-obese subjects. *Conclusion*. Higher secondhand smoke exposure measured objectively by serum cotinine levels was found to be associated with prehypertension in certain subgroups of a representative sample of the US population.

## 1. Introduction

Smoking is now recognized to be a major preventable risk factor for numerous outcomes, including cardiovascular diseases (CVD), and mortality [1–4]. Hypertension is a major public health problem affecting approximately 31.3% of US adults [5] and a well-recognized risk factor for CVD end-stage renal disease, and increased mortality. Several studies have reported an association between active smoking and hypertension among smokers [4, 6, 7]. However, it is not clear if, among nonsmokers, exposure to secondhand smoke (SHS) is a risk for increased blood pressure. There are only a few studies investigating the relationship between SHS and increased blood pressure in nonsmokers and all of these studies used self-reported exposure to SHS which is prone to misclassification [8–11]. 

Prehypertension, defined as systolic blood pressure (BP) ranging from 120–139 mmHg or diastolic BP ranging from 80–89 mmHg, is a preclinical stage in the high BP continuum, where subjects are at increased risk of developing hypertension in the near future [12]. From a prevention perspective, it is an important stage as an emerging body of the literature suggests that interventions at the prehypertension stage can prevent or delay the progression into established hypertension [13–18]. In this context, we examined the association between SHS exposure measured objectively by serum cotinine level and prehypertension among never smokers in a nationally representative sample of US adults after adjusting for age, sex, ethnicity, BMI, and other potential confounders. 

## 2. Methods 

The data for this study are derived from the National Health and Nutrition Examination Survey 2005–2008. Detailed description of NHANES study design and methods is available elsewhere [19, 20]. The NHANES included a stratified multistage probability sample representative of the civilian noninstitutionalized US population. Selection was based on counties, blocks, households, and individuals within households and included oversampling of non-Hispanic Blacks and Mexican Americans in order to provide stable estimates of these groups. 

Out of 20497 participants in NHANES 2005–2008, there were 10914 who were >20 years of age. We further excluded participants who were pregnant (*n* = 393), had prevalent cardiovascular disease (*n* = 1275), were former and current smokers (*n* = 4982), had hypertension defined as self-reported current BP-reducing medication use, and/or had systolic BP ≥140 mmHg and/or diastolic BP ≥90 mmHg (*n* = 1036), had missing or below the limit of detection (0.011 ng/mL) of serum cotinine level (*n* = 999), and those with missing information on systolic BP, diastolic BP, or other covariates (*n* = 202) included in the multivariable model. This resulted in 2027 participants who were included in the final analysis.

### 2.1. Main Outcome of Interest

#### 2.1.1. Prehypertension

Seated systolic and diastolic BPs were measured using a mercury sphygmomanometer according to the American Heart Association and JNC7 recommendations [21]. Up to 3 measurements were averaged for systolic and diastolic pressures. The outcome of interest in the current study was the presence of prehypertension, defined as systolic BP 120–139 mmHg systolic or diastolic BP 80–89 mmHg based on JNC7 criteria [22].

### 2.2. Exposure Measurements

Serum specimens were processed, stored, and shipped to the Division of Laboratory Sciences, National Center for Environmental Health, and Centers for Disease Control and Prevention for analysis. Detailed specimen collection and processing instructions were discussed in the NHANES Laboratory/Medical Technologists Procedures Manual (LPM). Serum cotinine was measured by isotope dilution high-performance liquid chromatography atmospheric pressure chemical ionization tandem mass spectrometry (ID HPLC-APCI MS/MS). Briefly, the serum sample was spiked with methyl-D3 cotinine as an internal standard, and after an equilibration period, the sample was applied to a basified solid-phase extraction column. Cotinine was extracted off the column with methylene chloride, the organic extract was concentrated, and the residue was injected onto a short C18 HPLC column. The eluant from these injections was monitored by APCI-MS/MS, and the *m*/*z* 80 daughter ion from the *m*/*z* 177 quasi-molecular ion was quantitated, along with additional ions for the internal standard, external standard, and for confirmation. Cotinine concentrations were derived from the ratio of native to labeled cotinine in the sample, by comparisons to a standard curve. Detailed description of serum cotinine measurement in NHANES is available online [20, 23]. Serum total cholesterol was measured enzymatically. Glycosylated hemoglobin measurements were performed on the Tosoh 2.2 Analyzer (Tosoh Medics, Inc., 347 Oyster Pt. Blvd., Suite 201, So. San Francisco, CA 94080, USA) in NHANES 2005-2006 and on the Automated HPLC System Glycohemoglobin Analyzer (Tosoh Medics, Inc., 347 Oyster Pt. Blvd., Suite 201, So.San Francisco, CA 94080, USA) in NHANES 2007-2008 at the University of Minnesota, Minneapolis, Minnesota, USA [20, 23].

Information on age, gender, race/ethnicity, alcohol intake (g/day), income, level of education, female hormone use, and cigarette smoking were obtained from a standardized questionnaire during a home interview. Income-poverty ratio was used as a measure of socioeconomic status. The Department of Health and Human Services' poverty guidelines were used to calculate this index. Poverty status was categorized into living below the federal poverty level (less than 1.00) and living at or above the federal poverty level (1.00 or more). Educational attainment was categorized into less than high school graduate, high school graduate, and more than high school graduate. Females who ever used estrogen or progesterone hormones, other than for birth control or fertility, were categorized into used/never used. Smoking status was categorized into never smokers (smoked <100 cigarettes during their lifetime), former smokers (smoked ≥100 cigarettes lifetime and currently not smoking), and current smokers (smoked ≥100 cigarettes lifetime and currently smoking) [19, 20]. Alcohol consumption was categorized into never drinking, former drinking, moderate (less than 3 drinks/day) and heavy drinker (3 or more drinks per day). Information on anthropometric, physical, and laboratory components were obtained during the medical examination center (MEC) examination. Body mass index (BMI) was calculated as weight in kilograms divided by height in meters squared.

### 2.3. Statistical Analysis

Serum cotinine levels were analyzed both as a continuous as well as a categorical variable. For analysis as a continuous variable, serum cotinine levels were log-transformed as a result of their skewed distribution. To examine the proportion of variance explained by including passive smoking, we calculated the modified *R* ^2^ values [24]. We categorized serum cotinine level into quartiles. We ran logistic regression models to calculate the odds ratio ((OR) and 95% confidence interval (CI)) of prehypertension for each higher serum cotinine quartile by using the lowest category as the referent. We ran two nested models, the age, sex adjusted model and the multivariable-adjusted model, additionally adjusting for ethnicity (non-Hispanic White, non-Hispanic Black, Mexican Americans and others), alcohol drinking (never drinker, former drinker, 1 or 2 drinks/day, ≥3 drinks/day), education (less than high school, high school, more than high school), poverty (living below the federal poverty level, living at or above the federal poverty level), use of female hormones (ever, never), BMI (normal weight, overweight, obese), glycosylated hemoglobin (%), and total cholesterol (mg/dL). To further ensure that the association is parallel for subgroups and rule out effect modification, we performed subgroup analyses by gender, race/ethnicity, and BMI categories. Trends in the OR of prehypertension across increasing serum cotinine levels were determined by modeling serum cotinine categories as an ordinal variable. Sample weights that account for the unequal probabilities of selection, oversampling, and nonresponse in the NHANES survey were applied for all analyses. Analyses were conducted using SAS (version 9.3, SAS Institute, Cary, NC, USA) software. Standard errors were estimated using the Taylor series linearization method.

## 3. Results

Table 1 presents the baseline characteristics of the study population, all of whom were never smokers. Among 2027 participants included in the study, approximately 54% were women and 62% were non-Hispanic White. Normal weight, overweight, and obese BMI categories were equally distributed. Prehypertension was present in 35.9% of the study population. The geometric mean of serum cotinine level was 0.1 ± 0.01 ng/mL. Compared with subjects who were included in the final study sample, those who were excluded owing to missing data were significantly more likely to be female and non-Hispanic White, but were similar with respect to other sociodemographic characteristics listed in Table 1.

Table 2 presents the association between serum cotinine levels and prehypertension. Serum cotinine levels were found to be positively associated with prehypertension in the age-, sex-adjusted model. Tests for linear trends for this association in the age, sex adjusted model as well as the multivariable-adjusted model were statistically significant. We performed an analysis where we calculated the modified *R* ^2^ from the multivariable logistic regression model. The *R* ^2^ in the multivariable model was 0.15 suggesting that 15% of the variance in the multivariable model for prehypertension was explained by serum cotinine. We also performed a supplementary analysis where we used serum cotinine (ng/mL) as a continuous variable. The multivariable odds ratio [95% C.I] of prehypertension was 1.039 [0.994, 1.085].

Next, to primarily examine confounding, we performed subgroup analyses by gender, race/ethnicity and BMI categories in Tables 3–5. In the subgroup analysis by gender (Table 3), the observed positive association between higher serum cotinine levels and prehypertension was mainly evident in men. In the subgroup analysis by race/ethnicity (Table 4), we observed that the positive association between higher serum cotinine levels and prehypertension was mainly present in non-whites, but not in whites. Finally, in the subgroup analysis by BMI (Table 5), we observed that the positive association between higher serum cotinine levels and prehypertension was mainly present in the nonobese group (BMI <30 kg/m^2^), but not in obese subjects (BMI ≥30 kg/m^2^). *P* interaction values for cross-product terms between cotinine levels X stratifying variables were all >0.2 except for gender (0.03).

We performed a supplementary analysis (shown in Table 6) where we confined the analysis to self-reported never smokers with serum cotinine levels <10 ng/mL; the results in this subgroup of low serum cotinine subjects were similar to our main findings in Table 2. 

## 4. Discussion

Among never smokers in the US general population who were free of hypertension, we initially found that higher levels of serum cotinine, an objective marker of SHS exposure, were positively associated with prehypertension independent of confounders. However, in subsequent subgroup analyses, the positive association was present only among men, but not women; among non-Whites, but not Whites; and among nonobese, but not obese subjects. 

Prehypertension is a preclinical stage where subjects are at increased risk of developing hypertension in the near future. Several previous studies have reported a positive association between smoking and hypertension [6, 7, 25–27]. An unintended consequence of smoking is exposure to SHS in nonsmokers. There are few studies investigating the relationship between SHS exposure and prehypertension among never smokers. A cross-sectional study involving clinically normotensive passive smokers found that self-reported passive smoking was associated with increased levels of BP in a dose-related manner [28]. In another study of 30 healthy nonsmoking women, systolic and diastolic BP were found to be acutely elevated following the exposure to passive smoking [9]. However, a limitation to these studies is that SHS exposure was assessed entirely on the basis of self-report without potential benefits of more objective measures, which in turn may have resulted in exposure misclassification.

In the current study, serum cotinine level was used to measure the level of exposure to SHS among never smokers. Cotinine is the principal metabolite of nicotine and has a 15–40 hr halflife [29]. Serum cotinine is considered a more precise measure of exposure to cigarette smoking when compared to self-reported smoking status [30, 31] and is considered an accurate biomarker of SHS exposure [32].

The exact mechanism underlying the observed association between SHS and prehypertension in never smokers remains unknown. A vasoconstriction mediated by nicotine is initially responsible for acute but transient increase in the systolic BP [33]. This phase is followed by a decrease in BP as a consequence of nicotine depressant effects [34]. In the long run, it has been suggested that carbon monoxide acts directly on the arterial wall potentially causing endothelial dysfunction [35–38] and structurally irreversible alterations such as arterial stiffness [11]. These changes potentially lead to a state of chronically elevated BP and prehypertension [10, 34]. 

In the current study, we performed subgroup analysis by gender, race/ethnicity, and BMI categories with the intent to examine confounding, a practice consistent with traditional methods of epidemiologic analysis. However, in the subgroup analysis by gender, post hoc, the positive association between serum cotinine and prehypertension was found to be present only in men, but not in women, suggesting possible gender differences. It is possible that hormonal differences in the way in which men and women metabolize nicotine may explain this observation. The main pathway of nicotine metabolism is, by oxidation, mediated by cytochrome P450 (CYP) and aldehyde oxidase enzymes [39]. It has been shown that women have increased lung expression of CYP enzymes compared with men which is related to estrogen [40, 41]. Consequently, accelerated breakdown of nicotine in the lungs may reduce circulating cotinine concentrations over time [42]. In addition, clearance of nicotine and cotinine was reported to be significantly higher in women than in men, especially in women taking oral contraceptives, indicating again that female hormones might play a key role in cotinine metabolism [39]. The underlying mechanism suggested for the effect of cotinine on blood pressure is through endothelial dysfunction and arterial stiffness, and, subsequently, higher levels of cotinine in the circulation may be related to higher endothelial dysfunction and eventually the development of prehypertension. Another suggested explanation is that differences in body fat percentage may contribute to such gender differences [43]. Women are known to have higher percentages of body fat [44–47]. Persons with relatively higher levels of body fat are likely to exhibit relatively lower cotinine activity in serum because of possible absorption of cotinine by fatty tissue [48]. This second explanatory hypothesis is also consistent with the current study's findings of no association between serum cotinine levels and prehypertension in the higher BMI group (BMI ≥30 kg/m^2^).

Even though the magnitude of odds ratios showed substantial differences by gender and BMI categories, the 95% confidence intervals were found to be overlapping. Therefore, it is possible that the differences we are observing are due to random variability and not true causal differences. Larger studies are needed to confirm if these observed differences in our study are statistically significant.

In a similar pattern, in the subgroup analysis by race/ethnicity, there was a significant positive association between serum cotinine levels and prehypertension in non-Whites, but not in Whites. Similar racial/ethnic differences were previously reported in the literature related to cotinine exposure levels. For example, Black smokers had higher cotinine levels than whites and Mexican Americans [49–51] possibly due to differences in brands used, inhalation pattern or ethnic differences in cotinine metabolism [50, 51]. It is also possible that there are differences in body fat percentage by race/ethnicity. For example, recent studies have shown that Whites have the highest body fat percentage for a given BMI [52].

In the current study, we were interested in the association between secondhand smoke exposure and prehypertension in never smokers. Although we included only participants who reported to be never smokers, some of these subjects had high serum cotinine levels, raising the possibility of misclassification bias with self-reported never smoking status. To address this concern, we performed a supplementary analysis shown in Table 6, limiting our analysis only to participants with serum cotinine <10 ng/mL. As expected, the results in this subgroup of low serum cotinine subjects were similar to our main findings, suggesting that misclassification due to self-reported smoking is not likely to bias our results.

This study has numerous strengths. Ours is the first study to date investigating the relationship between nicotine exposure measured objectively by serum cotinine level and prehypertension in never smokers. We believe that our use of serum cotinine will minimize the potential for misclassification bias. Moreover, the large national sample of racially and ethnically diverse US adults and the ability to adjust for numerous potential confounders add to the strengths of the study. The cross-sectional nature of NHANES represents the main limitation of the study as it does not allow us to draw conclusions regarding the causal role of nicotine exposure in prehypertension. 

In summary, this study provides evidence that SHS exposure measured by serum cotinine level in never smokers is associated with the prehypertension among adults free from hypertension in the general US population. In subgroup analyses, we also found that this association was evident in men, non-Whites, and nonobese subjects. If confirmed in prospective studies, our results suggest that SHS exposure may be a preventable factor for hypertension development in never smokers.

## Figures and Tables

**Table 1:** Baseline characteristics of the study population.

Characteristics	Mean values ± standard error (SE) or Sample size (weighted percentages)
Total sample size	2027
Women (%)	1109 (53.8%)
Age (years)	39.1 ± 0.5
Race/ethnicity (%)	
Non-Hispanic Whites	773 (61.9%)
Non-Hispanic Blacks	452 (12.6%)
Mexican Americans	484 (11.4%)
Others	318 (14.1%)
Education categories (%)	
Below high school	496 (15.3)
High school	467 (22.0)
Above high school	1064 (62.7)
Alcohol intake (%)	
Never drinker	358 (14.9%)
Former drinker	376 (16.4%)
Moderate drinker (1 or 2 drink/day)	815 (46.0%)
Heavy drinker (≥3 drinks/day)	478 (22.7%)
Body mass index (%)	
Normal weight (<25.0 kg/m^2^)	647 (35.2%)
Overweight (25.0–29.9 kg/m^2^)	709 (34.6%)
Obese (>30.0 kg/m^2^)	671 (30.2%)
Serum cotinine (ng/mL) (geometric mean)	0.1 ± 0.01
Glycosylated hemoglobin (%)	5.3 ± 0.02
Total cholesterol (mg/dL)	195.9 ± 1.2
Systolic blood pressure, mmHg	114.4 ± 0.3
Diastolic blood pressure, mmHg	69.1 ± 0.3
Below poverty level (%)	379 (12.2)
Prehypertension (%)	734 (35.9)

**Table 2:** Association between serum cotinine levels and prehypertension.

Cotinine quartiles	No. at risk	Cases	Age-, sex-adjusted odds ratio (95% confidence interval)	Multivariable-adjusted odds ratio (95% confidence interval)*
Quartile 1 (≤0.024 ng/mL)	488	165	1 (referent)	1 (referent)
Quartile 2 (0.025–0.054 ng/mL)	524	188	1.19 (0.89, 1.60)	1.19 (0.90, 1.58)
Quartile 3 (0.055–0.223 ng/mL)	508	185	1.44 (1.02, 2.02)	1.38 (0.97, 1.96 )
Quartile 4 (≥0.224 ng/mL)	507	196	1.53 (1.07, 2.19)	1.45 (1.00, 2.11)
*P* trend			0.0142	0.0451

*Adjusted for age (years), sex (men, women), ethnicity (non-Hispanic White, non-Hispanic Black, Mexican Americans, others), education categories (<high school, high school, >high school), drinking (never drinker, former drinker, 1 or 2 drink/day, ≥3 drinks/day), BMI (normal weight, overweight, obese), glycohemoglobin (%), total cholesterol (mg/dL), below poverty level (%), and hormone use (ever, never).

**Table 3:** Association between serum cotinine levels and prehypertension, by gender.

Serum cotinine quartiles	No. at risk	Cases	Age-adjusted odds ratio (95% CI)	Multivariable-adjusted odds ratio (95% CI)*
Men				
Quartile 1 (≤0.024 ng/mL)	192	74	1 (referent)	1 (referent)
Quartile 2 (0.025–0.054 ng/mL)	228	108	1.59 (1.00, 2.54)	1.62 (1.00, 2.62)
Quartile 3 (0.055–0.223 ng/mL)	225	105	1.85 (1.06, 3.22)	1.82 (1.02, 3.23)
Quartile 4 (≥0.224 ng/mL)	273	136	1.87 (1.15, 3.04)	1.80 (1.09, 2.98)
*P* trend			0.0209	0.0406
Women				
Quartile 1 (≤0.024 ng/mL)	296	91	1 (referent)	1 (referent)
Quartile 2 (0.025–0.054 ng/mL)	296	80	0.89 (0.58, 1.37)	0.86 (0.57, 1.29)
Quartile 3 (0.055–0.223 ng/mL)	283	80	1.23 (0.80, 1.92)	1.14 (0.71, 1.81)
Quartile 4 (≥0.224 ng/mL)	234	60	1.17 (0.75, 1.83)	1.02 (0.64, 1.63)
*P* trend			0.2413	0.6185

*Adjusted for age (years), ethnicity (non-Hispanic White, non-Hispanic Black, Mexican Americans, others), education categories (<high school, high school, >high school), drinking (never drinker, former drinker, 1 or 2 drink/day, ≥3 drinks/day), BMI (normal weight, overweight, obese), glycohemoglobin (%), total cholesterol (mg/dL), below poverty level (%), and hormone use (ever, never).

*P* interaction of cotinine quartiles and gender = 0.0303.

**Table 4:** Association between serum cotinine levels and prehypertension by ethnicity.

Serum cotinine quartiles	No. at risk	Cases	Age-, sex-adjusted odds ratio (95% CI)	Multivariable-adjusted odds ratio (95% CI)*
Whites				
Quartile 1 (≤0.024 ng/mL)	191	67	1 (referent)	1 (referent)
Quartile 2 (0.025–0.054 ng/mL)	205	78	1.16 (0.76, 1.78)	1.17 (0.76, 1.81)
Quartile 3 (0.055–0.223 ng/mL)	195	87	1.62 (1.03, 2.54)	1.49 (0.94, 2.37)
Quartile 4 (≥0.224 ng/mL)	182	73	1.38 (0.80, 2.37)	1.31 (0.75, 2.28)
*P*-trend			0.1267	0.2190
Non-Whites				
Quartile 1 (≤0.024 ng/mL)	297	98	1 (referent)	1 (referent)
Quartile 2 (0.025–0.054 ng/mL)	319	110	1.21 (0.82, 1.78)	1.22 (0.85, 1.75)
Quartile 3 (0.055–0.223 ng/mL)	313	98	1.15 (0.83, 1.61)	1.12 (0.79, 1.59)
Quartile 4 (≥0.224 ng/mL)	325	123	1.81 (1.27, 2.60)	1.76 (1.21, 2.56)
*P* trend			0.0043	0.0095

*Adjusted for age (years), sex (men, women), education categories (<high school, high school, >high school), drinking (never drinker, former drinker, 1 or 2 drink/day, ≥3 drinks/day), BMI (normal weight, overweight, obese), glycohemoglobin (%), total cholesterol (mg/dL), below poverty level (%), and hormone use (ever, never).

*P* interaction of cotinine quartiles and Whites = 0.5509.

**Table 5:** Association between serum cotinine levels and prehypertension by body mass index.

Serum cotinine quartiles	No. at risk	Cases	Age-, sex-adjusted odds ratio (95% CI)	Multivariable-adjusted odds ratio (95% CI)*
BMI < 30 kg/m^2^				
Quartile 1 (≤0.024 ng/mL)	350	105	1 (referent)	1 (referent)
Quartile 2 (0.025–0.054 ng/mL)	355	120	1.37 (0.98, 1.90)	1.38 (1.01, 1.89)
Quartile 3 (0.055–0.223 ng/mL)	326	106	1.79 (1.14, 2.81)	1.79 (1.13, 2.83)
Quartile 4 (≥0.224 ng/mL)	325	114	1.65 (1.07, 2.54)	1.66 (1.08, 2.55)
*P* trend			0.0163	0.0167
BMI ≥ 30 kg/m^2^				
Quartile 1 (≤0.024 ng/mL)	138	60	1 (Referent)	1 (Referent)
Quartile 2 (0.025–0.054 ng/mL)	169	68	0.85 (0.56, 1.29)	0.86 (0.57, 1.30)
Quartile 3 (0.055–0.223 ng/mL)	182	79	0.83 (0.48, 1.43)	0.84 (0.48, 1.47)
Quartile 4 (≥0.224 ng/mL)	182	82	1.07 (0.57, 2.00)	1.05 (0.53, 2.10)
*P* trend			0.8176	0.8858

*Adjusted for age (years), sex (men, women), ethnicity (non-Hispanic white, non-Hispanic black, Mexican Americans, others), education categories (<high school, high school, >high school), drinking (never drinker, former drinker, 1or 2 drink/day, ≥3 drinks/day), glycohemoglobin (%), total cholesterol (mg/dL), below poverty level (%), and hormone use (ever, never).

*P*-interaction of cotinine quartiles and obese = 0.3751.

**Table 6:** Association between serum cotinine levels and prehypertension among those with serum cotinine <10 ng/mL.

Serum cotinine quartiles	No. at risk	Prehypertension cases	Multivariable-adjusted odds ratio (95% confidence interval)*
Quartile 1 (≤0.024 ng/mL)	488	165	1 (referent)
Quartile 2 (0.025–0.054 ng/mL)	524	188	1.21 (0.91, 1.60)
Quartile 3 (0.055–0.223 ng/mL)	508	185	1.38 (0.97, 1.96)
Quartile 4 (≥0.224 ng/mL)	370	140	1.49 (1.06, 2.09)
*P* trend			0.0208

*Adjusted for age (years), sex (men, women), ethnicity (non-Hispanic White, non-Hispanic Black, Mexican Americans, others), education categories (<high school, high school, >high school), drinking (never drinker, former drinker, 1 or 2 drink/day, ≥3 drinks/day), BMI (normal weight, overweight, obese), glycohemoglobin (%), total cholesterol (mg/dL), below poverty level (%), and hormone use (ever, never).
